# Supplementary material for: Controlling the Heterodimerisation of the Phytosulfokine Receptor 1 (PSKR1) via Island Loop Modulation
Source: Int J Mol Sci. 2021 Feb 11;22(4):1806. doi: 10.3390/ijms22041806 (PMC7918699; doi:10.3390/ijms22041806)
Supplement: Supplementary file 1 [file ijms-22-01806-s001.pdf]

Supplementary data

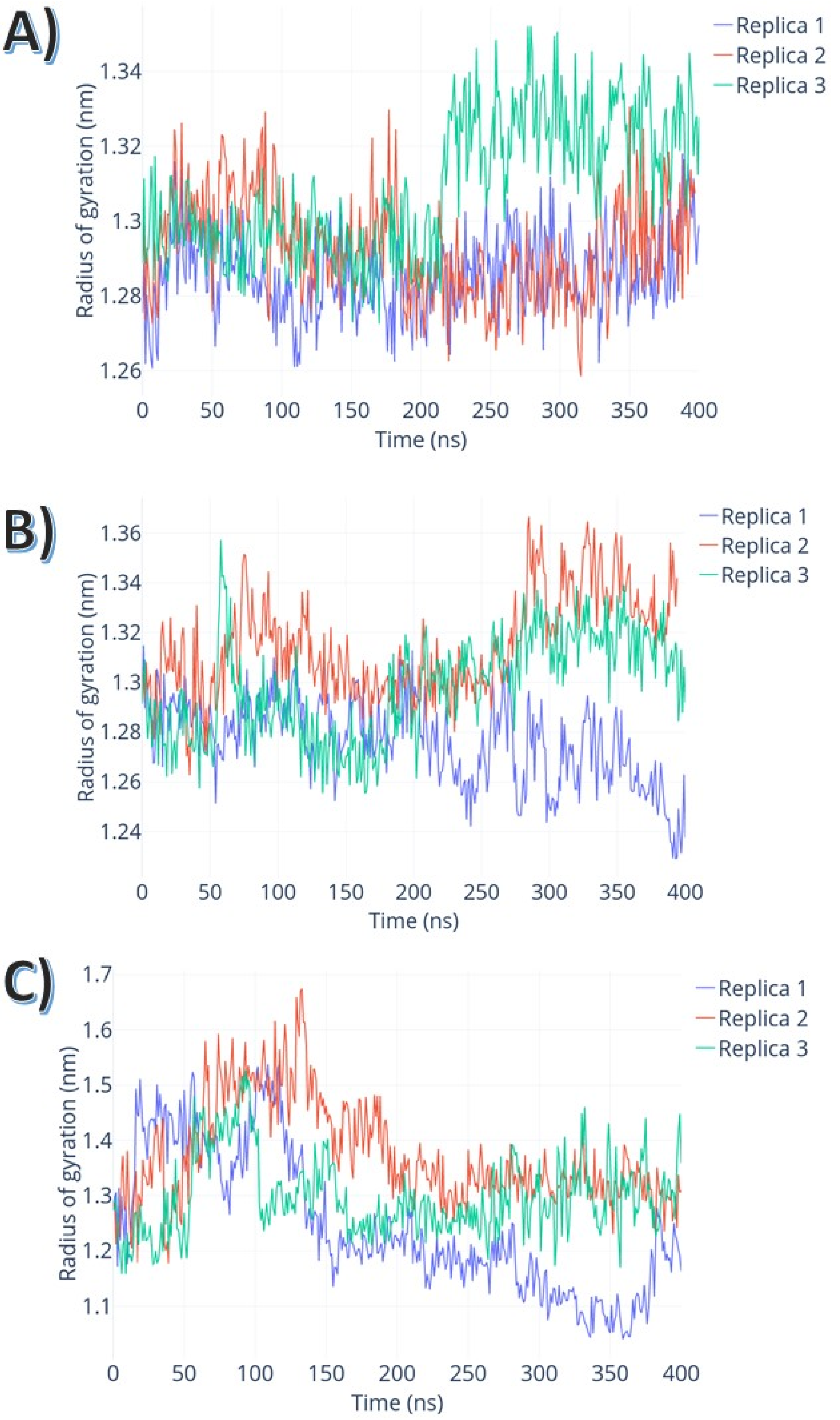

Supporting Figure 1: ID loop radius of gyration: A) PSKR1-PSK complex; B) Apo PSKR1; C) DcPSKR1

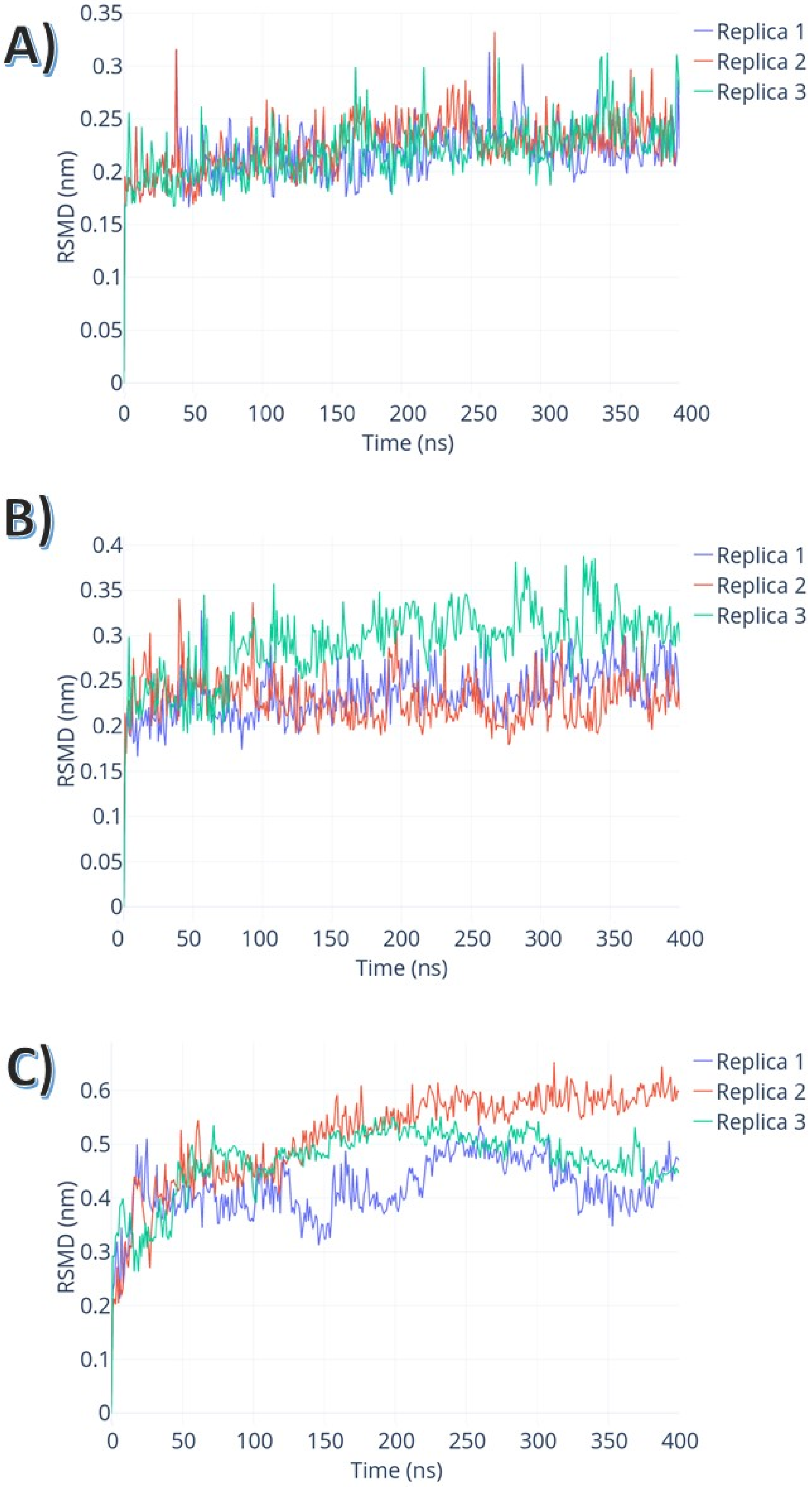

Supporting Figure 2: Root mean square deviation for all equilibrium runs: A) PSKR1-PSK complex; B) Apo PSKR1; C) DcPSKR1

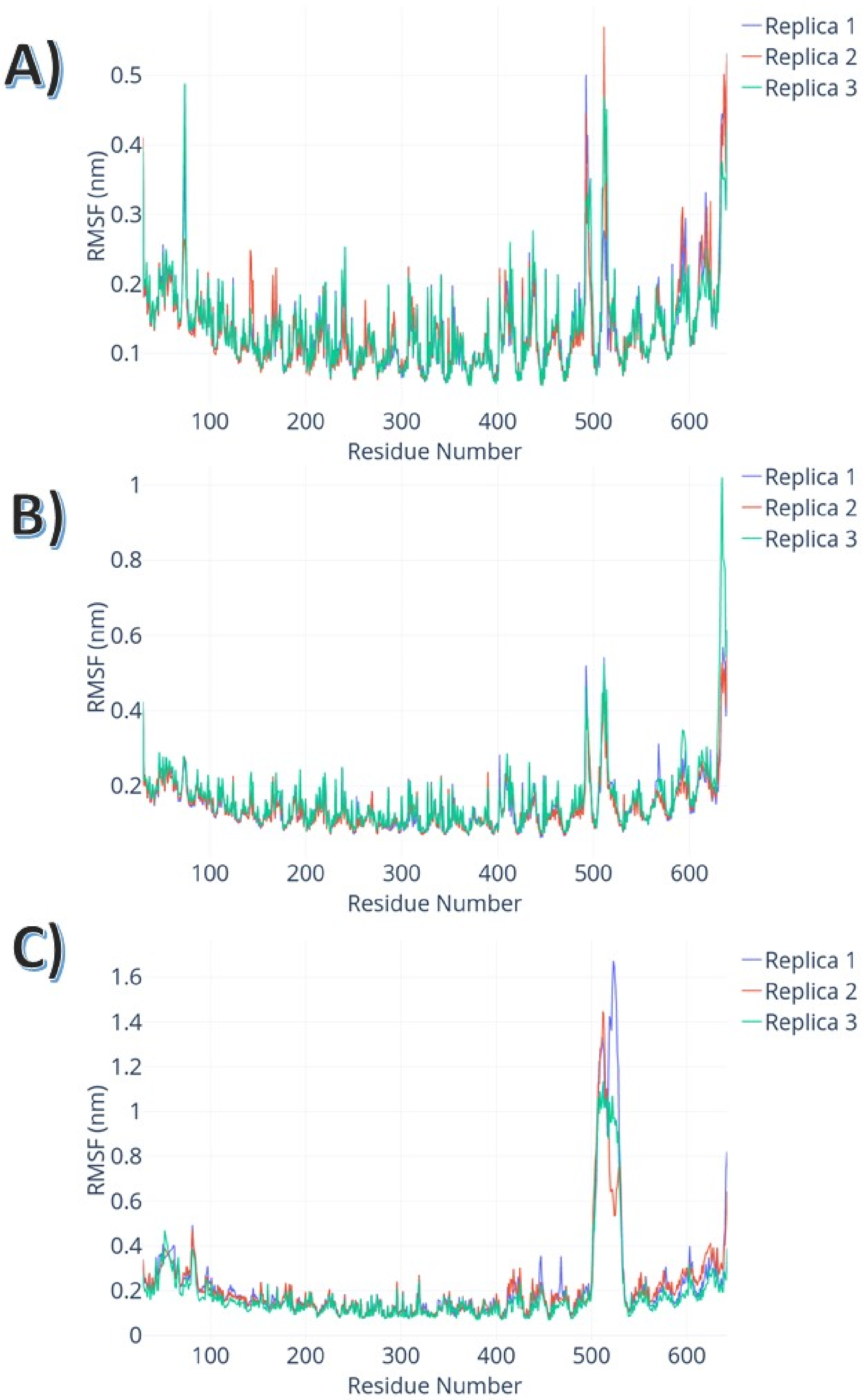

Supporting Figure 3: Residue root mean square fluctuation for all equilibrium runs: A) PSKR1-PSK complex; B) Apo PSKR1; C) DcPSKR1

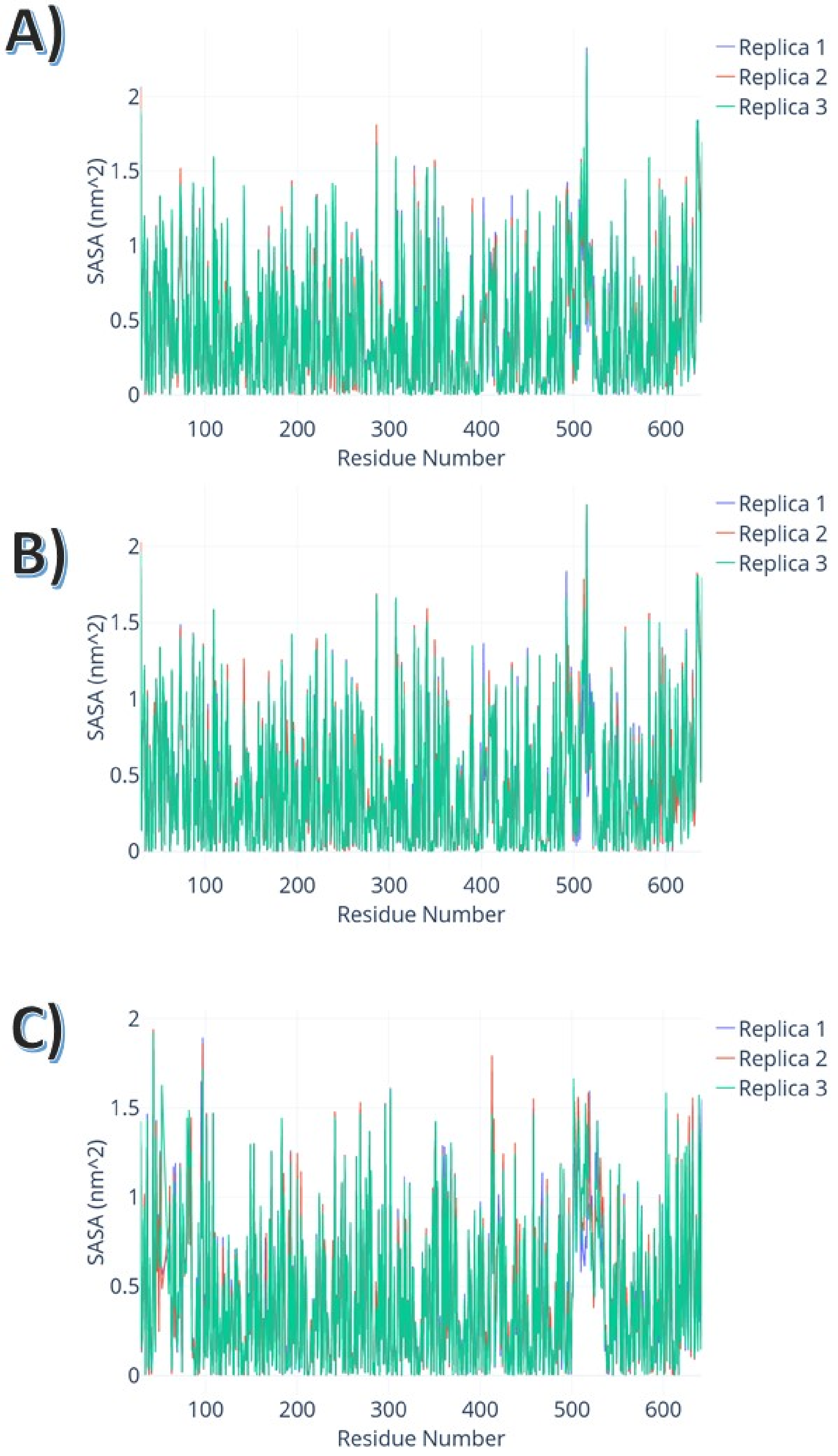

Supporting Figure 4: Solvent accessible surface area per residue for all equilibrium runs: A) PSKR1-PSK complex; B) Apo PSKR1; C) DcPSKR1

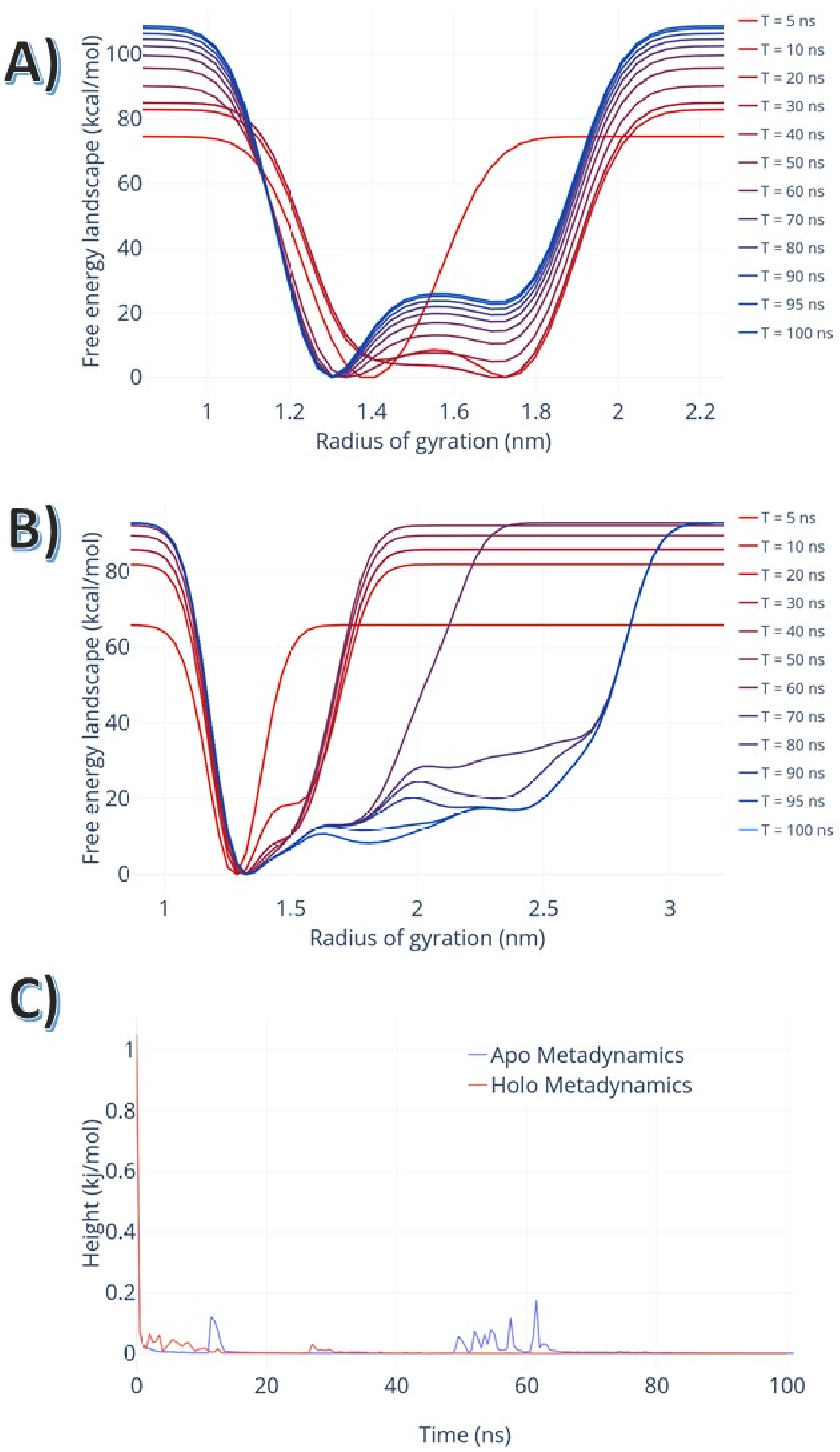

Supporting Figure 5: Well-tempered metadynamics convergence: A) PSKR1-PSK free energy landscape though time. B) Apo PSKR1 Free energy landscape through time. C) Gaussian height deposition for both systems through time.

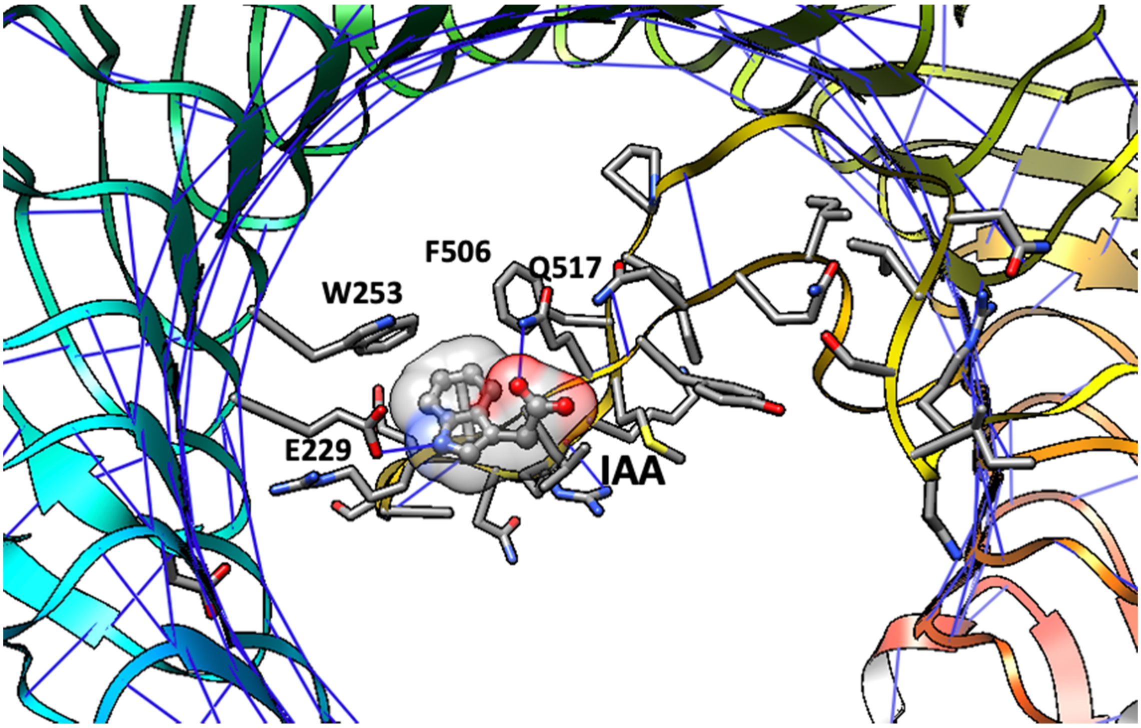

Supporting Figure 6: indole-3-acetic acid (IAA) binding mode to the ID loop. The ligand is labelled, coloured by heteroatom, and its molecular surface is highlighted. Its binding mode is stabilised by two H-bonds with E229 and Q517. Residues W253 and F506 contribute to favourable aromatic interactions between aromatic side chains and indole moiety of IAA. H-bonds are represented as blue lines. The protein is rendered as ribbon and coloured by gradient, from N-terminus (blue), to C-terminus (red). For the clarity, only side chains of the ID loop and those involved in IAA binding are showed.
